# Comprehensive analysis identifies cuproptosis-related gene DLAT as a potential prognostic and immunological biomarker in pancreatic adenocarcinoma

**DOI:** 10.1186/s12885-023-11042-7

**Published:** 2023-06-17

**Authors:** Xiaoling Zhang, Yuxin Zhou, Jiahe Hu, Xuefeng Yu, Haitao Xu, Zhichang Ba, Haoxin Zhang, Yanan Sun, Rongfang Wang, Xinlian Du, Ruishu Mou, Xuedong Li, Jiuxin Zhu, Rui Xie

**Affiliations:** 1 Department of Digestive Internal Medicine, Harbin Medical University Cancer Hospital, Harbin, 150081 China; 2 Department of Gastroenterological Surgery, Harbin Medical University Cancer Hospital, Harbin, 150081 China; 3 Department of Hepatobiliary and Pancreatic Surgery, Harbin Medical University Cancer Hospital, Harbin, 150081 China; 4 Medical Imaging Center, Harbin Medical University Cancer Hospital, Harbin, 150081 China; 5 Department of Pharmacology (National Key Laboratory of Frigid Zone Cardiovascular Diseases, State-Province Key Laboratories of Biomedicine-Pharmaceutics of China, Key Laboratory of Cardiovascular Medicine Research, Ministry of Education), College of Pharmacy, Harbin Medical University, Harbin, 150081 China

**Keywords:** Pancreatic adenocarcinoma, Cuproptosis, DLAT, Immunotherapy, Prognosis

## Abstract

**Background:**

Cuproptosis is a regulated cell death form associated with tumor progression, clinical outcomes, and immune response. However, the role of cuproptosis in pancreatic adenocarcinoma (PAAD) remains unclear. This study aims to investigate the implications of cuproptosis-related genes (CRGs) in PAAD by integrated bioinformatic methods and clinical validation.

**Methods:**

Gene expression data and clinical information were downloaded from UCSC Xena platform. We analyzed the expression, mutation, methylation, and correlations of CRGs in PAAD. Then, based on the expression profiles of CRGs, patients were divided into 3 groups by consensus clustering algorithm. Dihydrolipoamide acetyltransferase (DLAT) was chosen for further exploration, including prognostic analysis, co-expression analysis, functional enrichment analysis, and immune landscape analysis. The DLAT-based risk model was established by Cox and LASSO regression analysis in the training cohort, and then verified in the validation cohort. Quantitative reverse transcriptase polymerase chain reaction (RT-qPCR) and immunohistochemistry (IHC) assays were performed to examine the expression levels of DLAT in vitro and in vivo, respectively.

**Results:**

Most CRGs were highly expressed in PAAD. Among these genes, increased DLAT could serve as an independent risk factor for survival. Co-expression network and functional enrichment analysis indicated that DLAT was engaged in multiple tumor-related pathways. Moreover, DLAT expression was positively correlated with diverse immunological characteristics, such as immune cell infiltration, cancer-immunity cycle, immunotherapy-predicted pathways, and inhibitory immune checkpoints. Submap analysis demonstrated that DLAT-high patients were more responsive to immunotherapeutic agents. Notably, the DLAT-based risk score model possessed high accuracy in predicting prognosis. Finally, the upregulated expression of DLAT was verified by RT-qPCR and IHC assays.

**Conclusions:**

We developed a DLAT-based model to predict patients’ clinical outcomes and demonstrated that DLAT was a promising prognostic and immunological biomarker in PAAD, thereby providing a new possibility for tumor therapy.

**Supplementary Information:**

The online version contains supplementary material available at 10.1186/s12885-023-11042-7

## Introduction

Pancreatic cancer is a highly malignant tumor with extremely poor prognosis. In 2023, approximately 64,050 new cases will be detected, leading to estimated 50,550 deaths [1]. Pancreatic adenocarcinoma (PAAD) is the most common histological subtype of pancreatic cancer, accounting for approximately more than 85% of all cases [2]. The prevalence of PAAD is relevant to smoking, obesity, diabetes, alcohol use, chronic pancreatitis, and genetic factors [3]. Patients with PAAD are commonly unable to receive early diagnosis and treatments due to their non-specific symptoms and aggressive behaviors [4]. They generally result in poor outcomes with a 5-year survival rate as low as 9% [5]. At initial diagnosis, more than half of the patients suffer from metastatic diseases, exhibiting an average survival time of less than one year [6]. Multiple therapeutic approaches, such as surgery, chemotherapy, radiotherapy, targeted therapy, and immunotherapy have been developed to increase patients’ survival rates [7]. Nevertheless, PAAD continues to threaten human life and health seriously. Therefore, it is imperative to detect novel prognostic biomarkers to overcome the clinical dilemma in PAAD treatment.

Immunotherapy has provided more therapeutic opinions for cancer patients in recent years. Immune checkpoints inhibitors that target programmed cell death-1 (PD-1), programmed cell death-ligand 1 (PD-L1), and cytotoxic T-lymphocyte antigen-4 (CTLA-4) have been approved for the treatment of PAAD [8, 9]. Unfortunately, the immunosuppressive tumor microenvironment (TME) in PAAD that consists of dense fibrotic stromal, suppressed effector T cells, activated myeloid-derived suppressor cells, and insufficient secreted cytokines strongly limits the efficacy of immunotherapy [10]. Thus, it is crucial to identify useful immunological indicators to promote individualized immunotherapy in PAAD.

Cuproptosis, a newly defined cell death program, has attracted much attention due to its unique mechanism [11]. Unlike other cell death forms, such as apoptosis, autophagy, pyroptosis, and ferroptosis, cuproptosis is a copper-dependent pathway related to the tricarboxylic acid (TCA) cycle in mitochondria [12]. As an essential trace metal, copper is vital in regulating enzyme functions and body homeostasis. However, copper accumulation will induce cell death [13]. Tsvetkov et al. elucidated this lethal mechanism that excessive copper ions could directly bind to the lipoylated mitochondrial proteins, leading to lipoylated proteins aggregation and iron-sulfur cluster proteins reduction, eventually causing cell death [11]. Most researchers made explorations on the role of cuproptosis in tumors, and discovered that cuproptosis-related genes (CRGs) could function as prognostic biomarkers with the potential to guide immunotherapy in hepatocellular carcinoma [14], kidney cancer [15], breast cancer [16], and other tumors [17–19]. It was considered that cuproptosis might open the area of tumor treatment and provide new therapeutic targets in the future. Thus, we performed this analysis to explore the prognostic and therapeutic implications of cuproptosis in PAAD.

Herein, we conducted a comprehensive analysis to assess the function of CRGs in PAAD, including expression profiles, mutation and methylation status, correlation analysis, and consensus clustering analysis. We chose DLAT for further exploration and performed survival analysis to determine its prognostic value. To further investigate its biological function, we established the protein–protein interaction (PPI), gene–gene interaction, and co-expression network, then identified differentially expressed genes (DEGs) for enrichment analysis. We also uncovered the positive correlations between DLAT and immunological characteristics. In summary, our research indicated that DLAT could be a potential prognostic and immunological biomarker in PAAD.

## Materials and methods

### Data collection

Thirteen CRGs were obtained from the research of Tsvetkov et al., including 7 positive regulatory genes (FDX1, LIPT1, LIAS, DLD, DLAT, PDHA1, and PDHB), 3 negative regulatory genes (MTF1, GLS, and CDKN2A), and 3 copper transport-related genes (SLC31A1, ATP7A and ATP7B) [11]. The gene expression data of PAAD samples collected in the Cancer Genome Atlas (TCGA) database and normal samples saved in the genotype-tissue expression (GTEx) database were obtained from the UCSC Xena platform (http://xena.ucsc.edu/). The fragments per kilobase million (FPKM) sequencing data were transformed to transcripts per million (TPM) data and normalized by log2 (TPM + 1). Then, the TPM data of TCGA and GTEx databases were integrated using the "normalize between array" function of "limma" package (v3.52.4). To verify the differential expression of DLAT between tumor and normal tissues, GSE62452, GSE71729, GSE15471, and GSE16515 datasets were downloaded from the Gene Expression Omnibus (GEO) database (https://www.ncbi.nlm.nih.gov/geo/).

### Mutation and methylation analysis

Copy number variation (CNV) data were collected using the UCSC Xena platform. The location of CNV changes on chromosomes was visualized through "RCircos" package (v1.2.2). The promoter methylation levels of CRGs were analyzed by the UALCAN database (http://ualcan.path.uab.edu/) [20].

### Consensus clustering analysis

Patients in TCGA cohort were clustered using "ConsensusClusterPlus" package (v1.60.0) according to their expression levels of CRGs. In the consensus clustering analysis, the maximum number of clusters was defined as 6, and 80% of the total samples were drawn 1000 times, clusterAlg = "pam", innerLinkage = "ward.D". The optimal number of clusters was determined by consensus matrix heat map, consensus cumulative distribution function, and delta area plot together. Following that, we compared the age, gender, histologic grade, clinical stage, and survival status among different clusters. Survival differences were analyzed based on Kaplan–Meier curves using "survminer" package (v0.4.9). The "DESeq2" package (v1.36.0) was employed to identify DEGs between cluster A and cluster C on the basis of count data, with the threshold set at fold change > 2 and adjusted *p* < 0.05.

### Functional enrichment analysis

The "ClusterProfiler" package (v4.4.4) was utilized to explore the function of potential targets, including Gene Ontology (GO), Kyoto Encyclopedia of Genes and Genomes (KEGG) pathways [21], and Gene Set Enrichment Analysis (GSEA). The "HALLMARK" gene set was downloaded from the Molecular Signatures Database. The "GSVA" package (v1.44.5) was used to perform Gene Set Variation Analysis (GSVA) for analyzing pathways activities between different groups. The volcano map, box plot, and bubble plot were completed by "ggplot2" package (v3.3.6). The "pheatmap" package (v1.0.12) was used to draw the heatmap.

### Survival analysis

To assess the overall survival (OS) differences, we conducted Kaplan–Meier analysis based on log-rank test through "survival" package (v3.4–0) and "survminer" package (v0.4.9). The comparison of objective response rates between DLAT-high and DLAT-low groups was carried out by chi-square test. We then evaluated hazard ratio (HR) with 95% confidence interval (CI) using univariate and multivariate Cox proportional hazards regression. Forest plot was performed by "forestplot" package (v.3.0.0) to visualize *p* value, HR, and 95% CI of each variable.

### Construction of PPI, gene–gene interaction, and co-expression network

The STRING database (https://string-db.org/) was used to create the PPI network [22]. The GeneMANIA database (http://genemania.org/) was utilized to establish the gene–gene interaction network [23]. Pearson’s correlation analysis was applied to evaluate the correlations between DLAT and other genes for co-expression network construction.

### Immunological characteristics evaluation

Immunological characteristics were composed of immune cell infiltration, TME score, immunotherapy-predicted pathways, cancer-immunity cycle, and inhibitory immune checkpoints. We first analyzed the correlation between DLAT CNV and immune cell infiltration in TIMER database (https://cistrome.shinyapps.io/timer/) [24]. Immune infiltrating cells included B cells, CD8 + T cells, CD4 + T cells, macrophages, neutrophils, and dendritic cells. Following that, we evaluated the relationship between DLAT expression and the abundance of immune cells in the TIMER database. We also assessed the association between DLAT expression and the infiltration levels of 24 immune cells, which were generated by single sample Gene Set Enrichment Analysis (ssGSEA). The "IOBR" package (v0.99.9) was used to calculate the TME score [25].

The relationships between DLAT and immunotherapy-predicted pathways, as well as cancer-immunity cycle were also investigated. The 18 immunotherapy-predicted pathways included IFN-gamma signature, APM signal, base excision repair, cell cycle, DNA replication, Fanconi anemia pathway, and other pathways. The signature genes were acquired from previous studies [26, 27]. Moreover, the 7 steps of cancer-immunity cycle were described in 2013, including release of cancer antigens, cancer antigen presentation, priming and activation, trafficking of T cells to tumors, infiltration of T cells into tumors, recognition of cancer cells by T cells, and killing of cancer cells [28]. The signature gene set was obtained from the TIP website (http://biocc.hrbmu.edu.cn/TIP/) [29]. All the correlations were calculated through Pearson’s correlation analysis.

We integrated 5 frequently used immunological algorithms, including TIMER [30], CIBERSORT [31], MCPCOUNTER [32], QUANTISEQ [33], and EPIC [34] to obtain the immune cell infiltration matrix. The heatmap was carried out by "pheatmap" package (v1.0.12) to visualize the distribution of tumor-infiltrating immune cells.

Furthermore, we assessed the associations between DLAT and common inhibitory immune checkpoints, such as PD-L1, PD-L2, CTLA-4, TIM-3, TIGIT and so on. To investigate the role of DLAT in guiding immunotherapy, we analyzed the response to immune checkpoint blockade (ICB) by comparing our data with a meaningful melanoma dataset of 47 patients who received anti-PD-1 and anti-CTLA-4 treatments by subclass mapping algorithm [35–37]. Moreover, IMvigor210 [27], CheckMate [38], GSE78220 [39], and GSE91061 [40] cohorts were also used to predict the clinical prognosis of patients under immunotherapy.

### Construction of risk score model

The "DESeq2" package (v1.36.0) was used to determine the DEGs (fold change = 1.5, adjusted *p* < 0.05) between DLAT-high and DLAT-low groups. Next, we conducted the univariable Cox regression analysis to obtain the DEGs with prognostic value. The risk score model was then developed on the basis of the prognostic DEGs. The "survival" package (v3.4–0) and "glmnet" package (v4.1–4) were employed to perform Cox and LASSO regression analysis, respectively. All patients in the TCGA-PAAD cohort were included in the training set. They were classified into low- or high-risk groups using the median risk score. Survival differences were evaluated by log-rank test. The accuracy of model was estimated by time-dependent receiver operating characteristic (ROC) curves with corresponding areas under the curve (AUC) values using "survivalROC" package (v1.0.3).

### Cell culture

Human pancreatic cancer cell lines (BxPC-3 and PANC-1) and normal pancreatic cell line (HPDE6-C7) were obtained from the Chinese Academy of Sciences (Shanghai, China) and cultured with complete growth medium, as recommended by the manufacturer. All cells were incubated at 37℃ in a 5% of CO_2_ environment.

### Quantitative reverse transcriptase polymerase chain reaction (RT-qPCR)

Total RNA was extracted of cells using TRIzol reagent. Next, the RNA was reverse transcribed into cDNA using a PrimeScript RT Reagent Kit with gDNA Eraser (Takara, Japan) according to the manufacturer’s instructions. RT-qPCR was conducted using TB Green Premix Ex Taq II (Takara, Japan). The reaction conditions were set as follows: 95℃ for 30 s, 40 cycles of 95 ℃ for 3 s and 60 ℃ for 30 s. Relative mRNA levels were determined by the 2^−∆∆Ct^ method using GAPDH mRNA as an internal control. The primer sequences were shown as follows. DLAT-forward: ACTCCCCAGCCTTTAGCTC, DLAT-reverse: CAATCCCTTTCTCTACTGCCAAC, GAPDH forward: GCTGAGAACGGGAAGCTTGT, GAPDH reverse: GCCAGGGGTGCTAAGCAGTT.

### Immunohistochemistry (IHC)

Human pancreatic cancer tissues were collected from 12 patients in Harbin Medical University Cancer Hospital. Patients have not received any anti-cancer treatment before tissue collection. Tissues were fixed in formalin, embedded in paraffin, and cut into 3 μm sections. The 2-step plus Poly-HRP Anti-Rabbit IgG detection system with DAB solution (Elabscience, E-IR-R217) was used to perform IHC staining. Sections were incubated with primary antibody against DLAT (1:500, Proteintech, 13,426-1AP) overnight at 4℃, followed by secondary antibody polyperoxidase-anti-rabbit IgG for 30 min at 37℃, and finally stained by DAB reagent.

### Statistical analysis

The comparison between 2 groups was analyzed by Wilcoxon test, and the comparison among 3 groups was evaluated by Kruskal–Wallis test. Chi-square test was used for the comparison of objective response rate. Kaplan–Meier analysis was utilized to assess the survival differences through log-rank test. Pearson’s correlation analysis was employed to examine the correlation between 2 variables. All statistical analyses were performed using R (v4.2.1). *p* < 0.05 was considered statistically significant.

## Results

### Expression profiles and mutation status of CRGs in PAAD

**Fig. 1:**
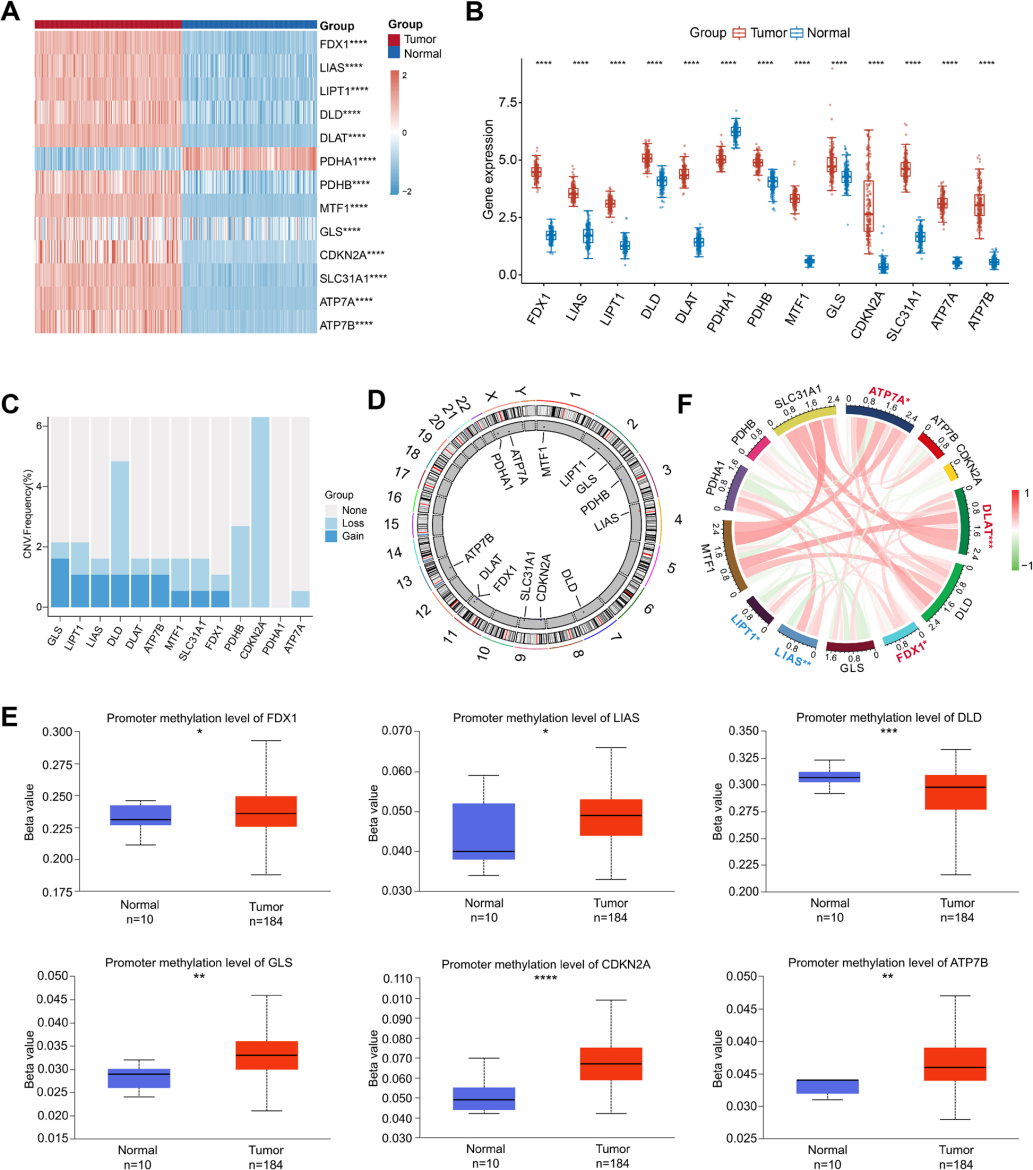
Characteristics of CRGs in PAAD. **A** Heat map and **B** box plot of 13 CRGs in tumor tissues and normal tissues. **C** CNV frequency of CRGs in PAAD. **D** Location of CNV alterations on chromosomes. **E** Promoter methylation levels of CRGs in tumor tissues and normal tissues. **F** Correlation analysis and prognostic values of CRGs in PAAD. Positive correlation, red; negative correlation, green (*p < 0.05; **p < 0.01; ***p < 0.001; ****p < 0.0001)

Tsvetkov et al. have defined 13 CRGs which were expected to serve as novel targets in cancer treatment. We analyzed their expression levels in PAAD tissues and normal tissues, and found that all genes were differently expressed (Fig. 1A-B). In detail, FDX1, LIAS, LIPT1, DLD, DLAT, PDHB, MTF1, GLS, CDKN2A, SLC31A1, ATP7A, and ATP7B were highly expressed in tumor tissues, while PDHA1 was upregulated in normal tissues. Moreover, we examined the CNV alterations and revealed that GLS had the most significant copy number gain, whereas CDKN2A showed the most significant copy number loss (Fig. 1C). The location of CNV alterations on chromosomes was visualized in Fig. 1D. Promoter methylation levels of FDX1, LIAS, GLS, CDKN2A, and ATP7B were upregulated in tumor tissues, whereas the level of DLD was upregulated in healthy tissues (Fig. 1E). In addition, most CRGs were positively correlated with each other (Fig. 1F). The log-rank analysis depicted 5 prognostic genes, of which FDX1, DLAT, and ATP7A were associated with poor outcomes, while LIAS and LIPT1 were related to favorable outcomes (Fig. 1F).

### Identification and biological characteristics of CRG clusters in PAAD

**Fig. 2:**
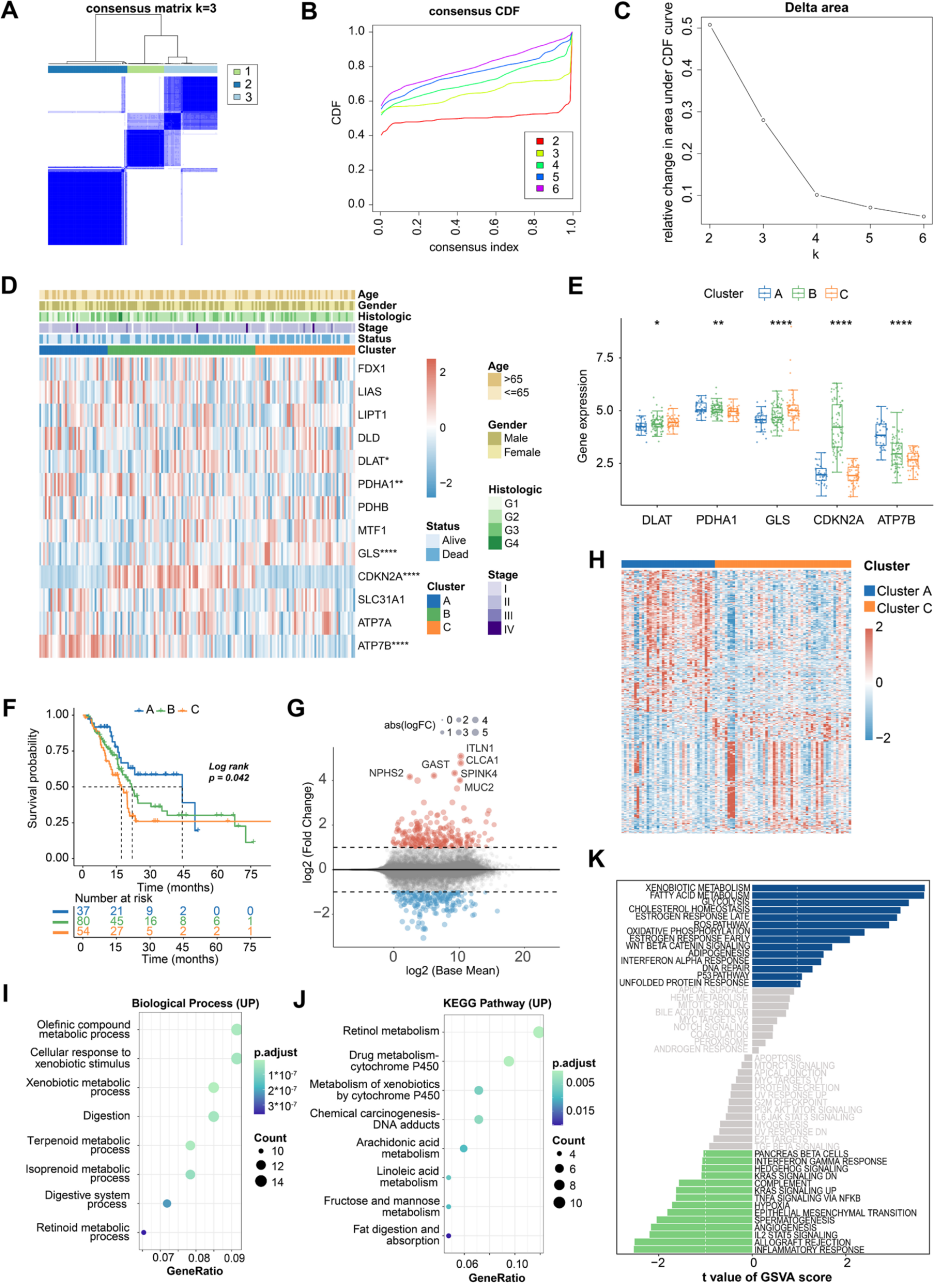
Consensus clustering analysis of CRGs in PAAD. **A-C** Consensus matrix heatmap defining 3 clusters and their correlation area. **D** Associations between 3 clusters and clinical features. **E** Box plot of 5 differentially expressed CRGs among 3 clusters. **F** Kaplan–Meier curves for patients in different subgroups. **G** Volcano plot and **H** heatmap of DEGs between cluster A and C. **I** GO, **J** KEGG, and **K** GSVA of upregulated DEGs between cluster A and C (*p < 0.05; **p < 0.01; ****p < 0.0001)

Based on the expression of CRGs, patients were clustered using consensus clustering algorithm. The results showed that consensus matrix k = 3 can produce the best result (Fig. 2A-C). Patients were classified into 3 subgroups, termed cluster A (*n =* 37), cluster B (*n =* 80), and cluster C (*n =* 54). The associations between 3 clusters and clinical features were presented in Fig. 2D. Five genes (DLAT, PDHA1, GLS, CDKN2A, and ATP7B) were differentially expressed among clusters. PDHA1 and ATP7B were increased in cluster A, CDKN2A was upregulated in cluster B, while DLAT and GLS were highly expressed in cluster C (Fig. 2E). Survival analysis indicated a significant difference on OS (log-rank test, *p* = 0.042) (Fig. 2F). Patients in cluster A exhibited the best median OS of 44.4 months, whereas those in cluster C showed the worst OS of 17.2 months, implying that patients in cluster C had higher degree of malignancy than those in cluster A.

We identified 224 upregulated genes (including ITLN1, CLCA1, SPINK4, NPHS2, GAST, and MUC2) and 194 downregulated genes between cluster A and cluster C (Fig. 2G-H). To explore their function, we conducted the enrichment analysis based on upregulated DEGs. The results of GO, KEGG, and GSVA together uncovered that upregulated DEGs were closely associated with tumor metabolic regulation, such as olefinic compound metabolic process, retinol metabolism, and xenobiotic metabolism (Fig. 2I-K).

### Expression profiles and prognostic values of DLAT in PAAD

In this research, DLAT possessed the lowest *p* value (*p* < 0.001) in prognostic analysis, indicating that DLAT was strongly associated with clinical outcomes in PAAD. (Fig. 1F). Moreover, we found that the survival time was increasingly shortened in cluster A, B, and C (Fig. 2F). Meanwhile, the expression levels of DLAT were getting higher (Fig. 2E). This result further suggested that high expression of DLAT was correlated with poor prognosis. Thus, we selected DLAT for further analysis.

**Fig. 3:**
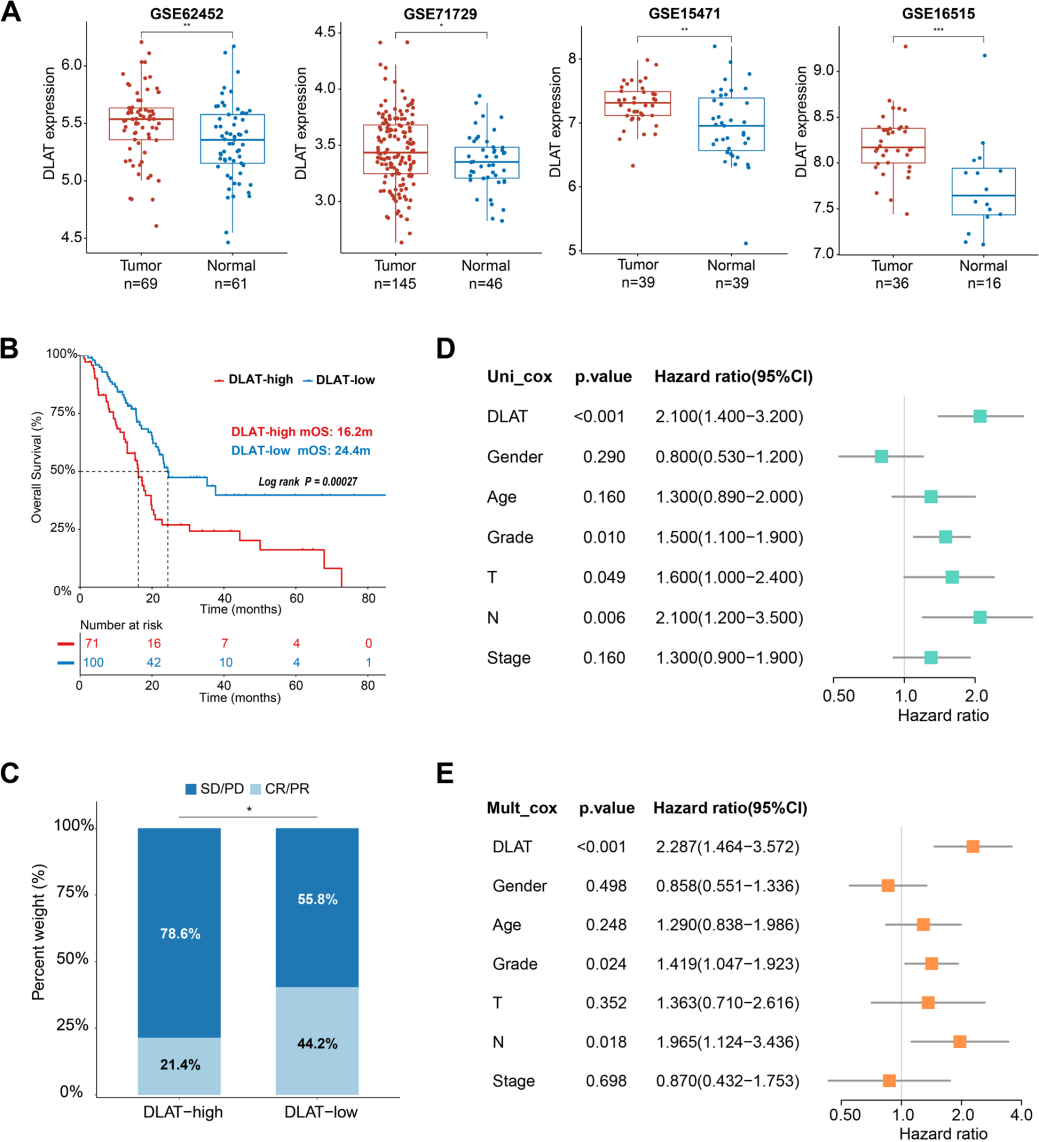
Expression profiles and prognostic values of DLAT in PAAD. **A** Box plot of DLAT expression between tumor and normal tissues in GSE62452, GSE71729, GSE15471, and GSE16515 datasets. **B** Kaplan–Meier curves for patients in DLAT-high and DLAT-low groups. **C** Comparison of clinical response rates (CR, complete response; PR, partial response; SD, stable disease; PD, progressive disease) in DLAT-high and DLAT-low groups. **D** Univariate and **E** multivariate Cox analysis of DLAT expression and clinical factors (*p < 0.05; **p < 0.01; ***p < 0.001)

We downloaded the external datasets GSE62452, GSE71729, GSE15471, and GSE16515 to confirm the differential expression of DLAT. In accordance with previous results, DLAT was significantly increased in tumor tissues than normal tissues (Fig. 3A). To further examine its prognostic signification, patients in TCGA cohort with the survival time of more than 30 days were separated into DLAT-high (*n =* 71) and DLAT-low (*n =* 100) groups. We found that DLAT-high patients exhibited a shorter OS (log-rank test, *p* < 0.001) (Fig. 3B) and a lower objective response rate (chi-square test, *p* = 0.043) (Fig. 3C) than DLAT-low patients. Moreover, increased DLAT expression was considered as an independent risk factor for survival (univariable Cox, HR = 2.100, *p* < 0.001; multivariable Cox, HR = 2.287, *p* < 0.001) (Fig. 3D-E).

### Construction of PPI, gene–gene interaction, and co-expression network

**Fig. 4:**
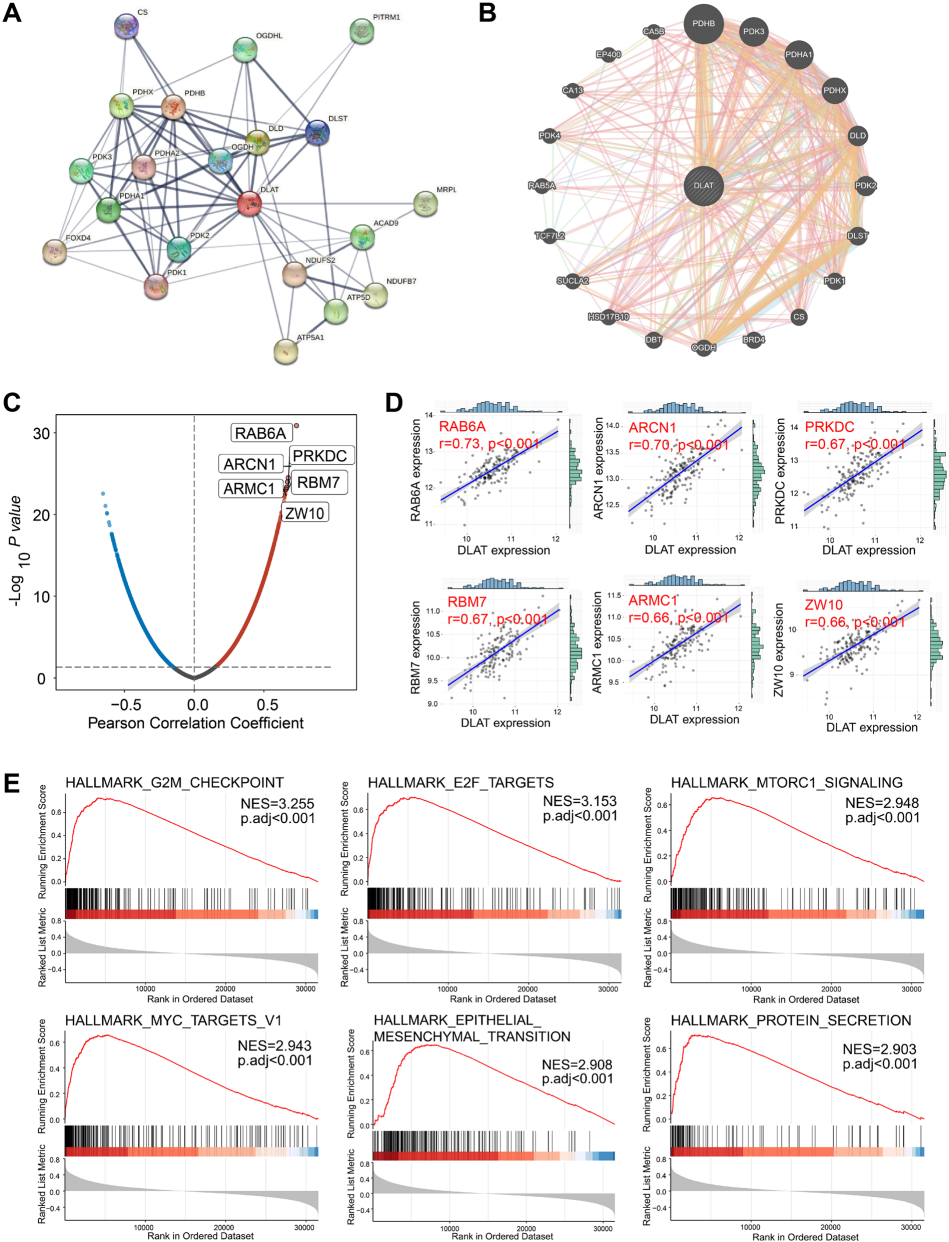
Integrated analysis of PPI, gene–gene interaction, and co-expression network. **A** Construction of PPI network. **B** Construction of gene–gene interaction network. **C** Volcano map of co-expression genes associated with DLAT. **D** Correlation analysis of DLAT expression with RAB6A, ARCN1, PRKDC, RBM7, ARMC1, and ZW10. **E** Hallmark terms enriched in G2/M checkpoint, E2F targets, mTORC1 signaling, MYC targets, epithelial-mesenchymal transition, and protein secretion based on GSEA

In order to analyze the biological function of DLAT, we first established the PPI network in the STRING database (Fig. 4A). Then, the DLAT-associated gene–gene interaction network was built using the GeneMANIA database (Fig. 4B). It was observed that the CRGs, including PDHB, PDHA1, and DLD were involved in the PPI and gene–gene interaction network. Additionally, we performed correlation analysis to construct DLAT co-expression network. A total of 5474 genes were found to be positively related to DLAT (Fig. 4C) (Supplementary Table 1). Among them, 6 genes exhibited the strongest correlation, namely RAB6A (r = 0.73, *p* < 0.001), ARCN1 (r = 0.70, *p* < 0.001), PRKDC (r = 0.67, *p* < 0.001), RBM7 (r = 0.67, *p* < 0.001), ARMC1 (r = 0.66, *p* < 0.001), and ZW10 (r = 0.66, *p* < 0.001) (Fig. 4D). GSEA of DLAT co-expression network indicated the top 6 enriched hallmark terms, including G2/M checkpoint (NES = 3.255, *p* < 0.001), E2F targets (NES = 3.153, *p* < 0.001), mTORC1 signaling (NES = 2.948, *p* < 0.001), MYC targets (NES = 2.943, *p* < 0.001), epithelial-mesenchymal transition (NES = 2.908, *p* < 0.001), and protein secretion (NES = 2.903, *p* < 0.001), which were associated with cell proliferation and tumor metastasis (Fig. 4E). The detailed description of GSEA was shown in Supplementary Table 2.

### Identification of DEGs and enrichment analysis

**Fig. 5:**
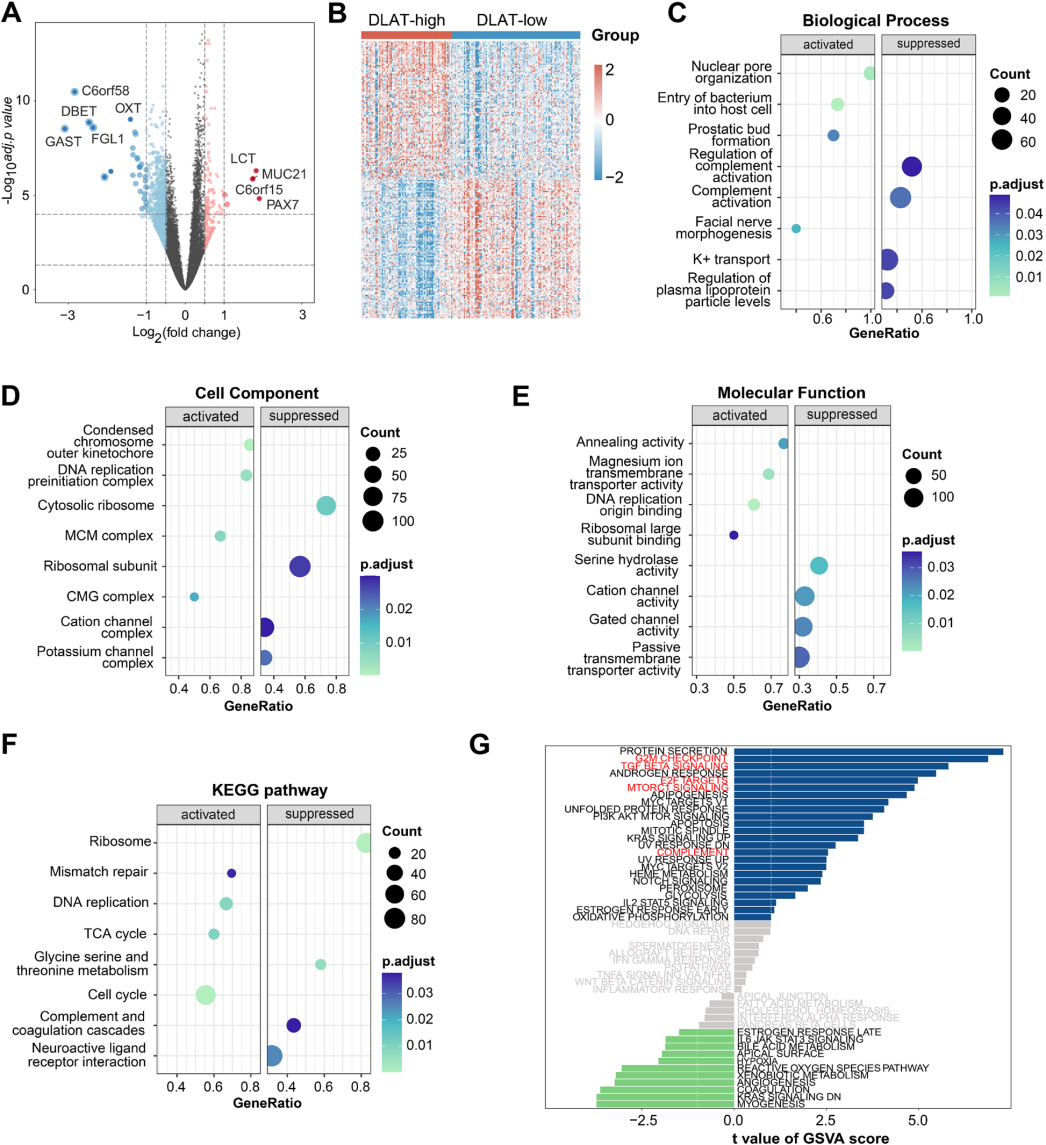
Identification of DEGs between DLAT-high and DLAT-low groups and functional enrichment analysis. **A** Volcano plot of DEGs. **B** Heatmap plot of the top 200 DEGs. **C-F** GO and KEGG pathway analysis of DEGs. **G** Differences in pathway activities scored per patient by GSVA between DLAT-high and DLAT-low groups

We identified 144 upregulated genes (such as LCT, MUC21, PAX7, and C6orf15) and 680 downregulated genes (such as C6orf58, GAST, DBET, FGL1, and OXT) in DLAT-high group when compared with DLAT-low group (Fig. 5A) (Supplementary Table 3). The top 200 DEGs were shown in Fig. 5B. We then carried out enrichment analysis to clarify the function of DLAT. GO analysis consisted of biological process, cell component, and molecular function. The results showed that DEGs were involved in nuclear pore organization, condensed chromosome outer kinetochore, and annealing activity (Fig. 5C-E). KEGG analysis demonstrated that DEGs were primarily engaged in cell proliferation and DNA repair, such as mismatch repair, DNA replication, and cell cycle (Fig. 5F). The following GSVA revealed the enrichments in G2/M checkpoint, E2F targets, mTORC1 signaling, TGF-β signaling, and complement, which were related to cell proliferation and immune response (Fig. 5G). In conclusion, DLAT may promote the tumor progression through these tumor-related pathways.

### Immunological analysis of DLAT in PAAD

**Fig. 6:**
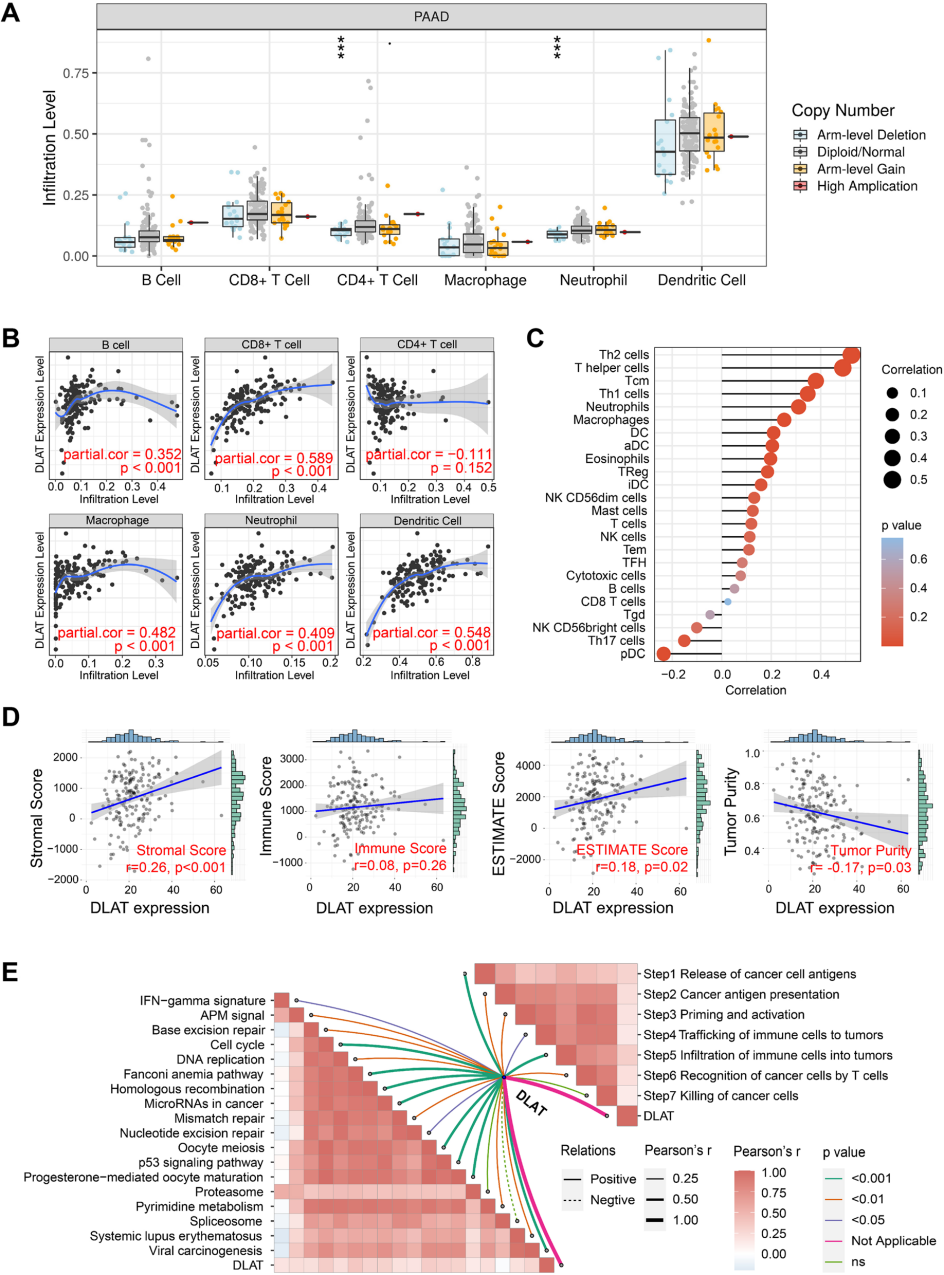
Correlation analysis between DLAT expression and immune cell infiltration. **A** Immune cell infiltration under various copy numbers of DLAT. **B** Correlations between DLAT and immune cell infiltration based on TIMER database. **C** Correlations between DLAT and immune cell infiltration calculated by ssGSEA algorithm. **D** Correlations between DLAT and stromal, immune, ESTIMATE scores, as well as tumor purity. **E** Correlations between DLAT and immunotherapy-predicted pathways (left) and cancer-immunity cycle (right) (***p < 0.001)

We first investigated the impact of DLAT on immune cell infiltration in the TIMER database, and observed that DLAT CNV has a significant association with the infiltration levels of CD4 + T cell and neutrophil (Fig. 6A). Meanwhile, DLAT expression was positively correlated with the infiltration levels of B cell (r = 0.352, *p* < 0.001), CD8 + T cell (r = 0.589, *p* < 0.001), macrophage (r = 0.482, *p* < 0.001), neutrophil (r = 0.409, *p* < 0.001), and dendritic cell (r = 0.548, *p* < 0.001) (Fig. 6B). In addition, DLAT expression exhibited positive correlations with most immune cells analyzed by ssGSEA algorithm (Fig. 6C). We then calculated the TME scores based on "ESTIMATE" package, and revealed close associations between DLAT and stromal score (r = 0.26, *p* < 0.001), ESTIMATE score (r = 0.18, *p* = 0.02), as well as tumor purity (r = -0.17, *p* = 0.03) (Fig. 6D). Moreover, DLAT was positively related to steps 1–6 of cancer-immunity cycle and the enrichment scores of immunotherapy-predicted pathways gene signatures, such as cell cycle, Fanconi anemia pathway, homologous recombination, and others (Fig. 6E).

**Fig. 7:**
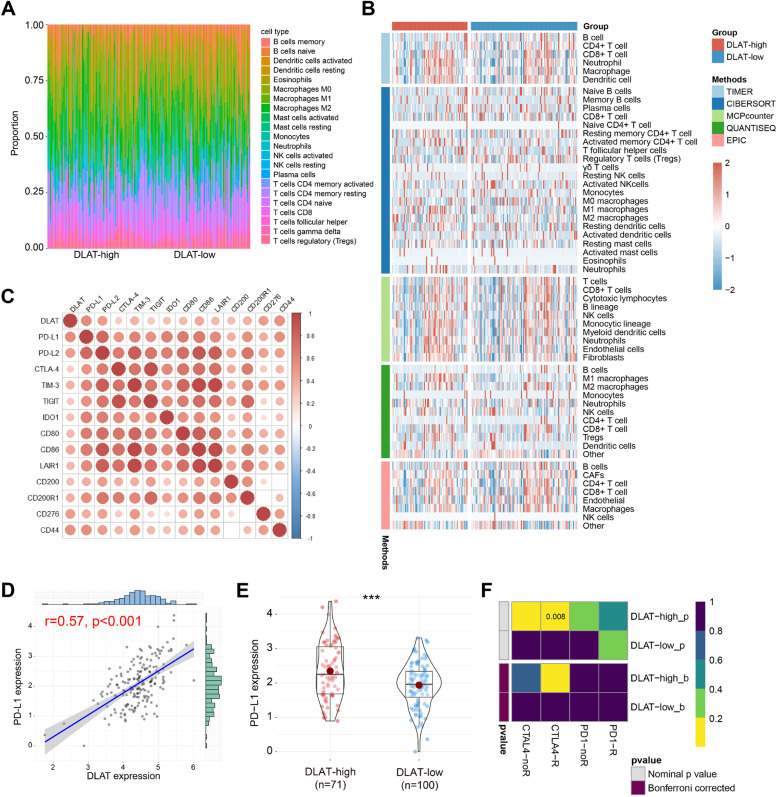
Immunological characteristics in DLAT-high and DLAT-low groups. **A** Proportions of 22 immune cell types in DLAT-high and DLAT-low groups. **B** Heatmap of immune cell infiltration in DLAT-high and DLAT-low groups. **C** Correlations between DLAT and inhibitory immune checkpoints. **D** Correlations between DLAT and PD-L1 expression. **E** Comparison of PD-L1 expression in DLAT-high and DLAT-low groups. **F** Submap algorithm evaluated the expression similarity between TCGA-PAAD patients and melanoma patients receiving anti-PD-1 and anti-CTLA-4 therapies (R represents responsive, while noR represents no responsive; ***p < 0.001)

According to previous results, DLAT was likely to produce a favorable effect on anticancer immune response in PAAD. Immune cell infiltration in DLAT-high and DLAT-low groups was shown in Fig. 7A. We integrated 5 main stream immunoinformatic methods, including TIMER, CIBERSORT, MCPCOUNTER, QUANTISEQ, and EPIC, to elucidate tumor-infiltrating immune cell types in different groups. The infiltration levels of immune cells were different between DLAT-high and DLAT-low groups (Fig. 7B).

Immune checkpoints played an immunosuppressive role in immune response by inhibiting immune cell activation. In this research, we found that DLAT expression was positively related to common inhibitory immune checkpoints, such as PD-L1, PD-L2, CTLA-4, TIM-3, and TIGIT (Fig. 7C). PD-L1 expression was an important biomarker to predict immunotherapy response. We, therefore, examined the relationship between DLAT and PD-L1, and demonstrated a positive correlation between them (r = 0.57, *p* < 0.001) (Fig. 7D). Compared to DLAT-low group, PD-L1 was statistically upregulated in DLAT-high group (Fig. 7E). Subsequently, we compared the expression profile of TCGA-PAAD patients with a melanoma dataset containing 47 patients who received anti-PD-1 and anti-CTLA-4 treatments [35–37]. It was observed that DLAT-high patients were more responsive to anti-CTLA-4 therapy than DLAT-low patients (Nominal* p* = 0.008) (Fig. 7F). In addition, patients in DLAT-high group were more promising to respond to anti-PD-L1 therapy (Supplementary Fig. 1A). However, when comparing the expression profile of DLAT-high group with PD-1-response group, the results showed insignificant differences, indicating that DLAT exhibited little impact on the anti-PD-1 therapeutic response (Supplementary Fig. 1B-D). The diverse results may be attributed to the stronger correlation between DLAT and PD-L1/CTLA-4 than it between DLAT and PD-1 (Supplementary Fig. 1E).

### Development and validation of DLAT-based risk score model

**Fig. 8:**
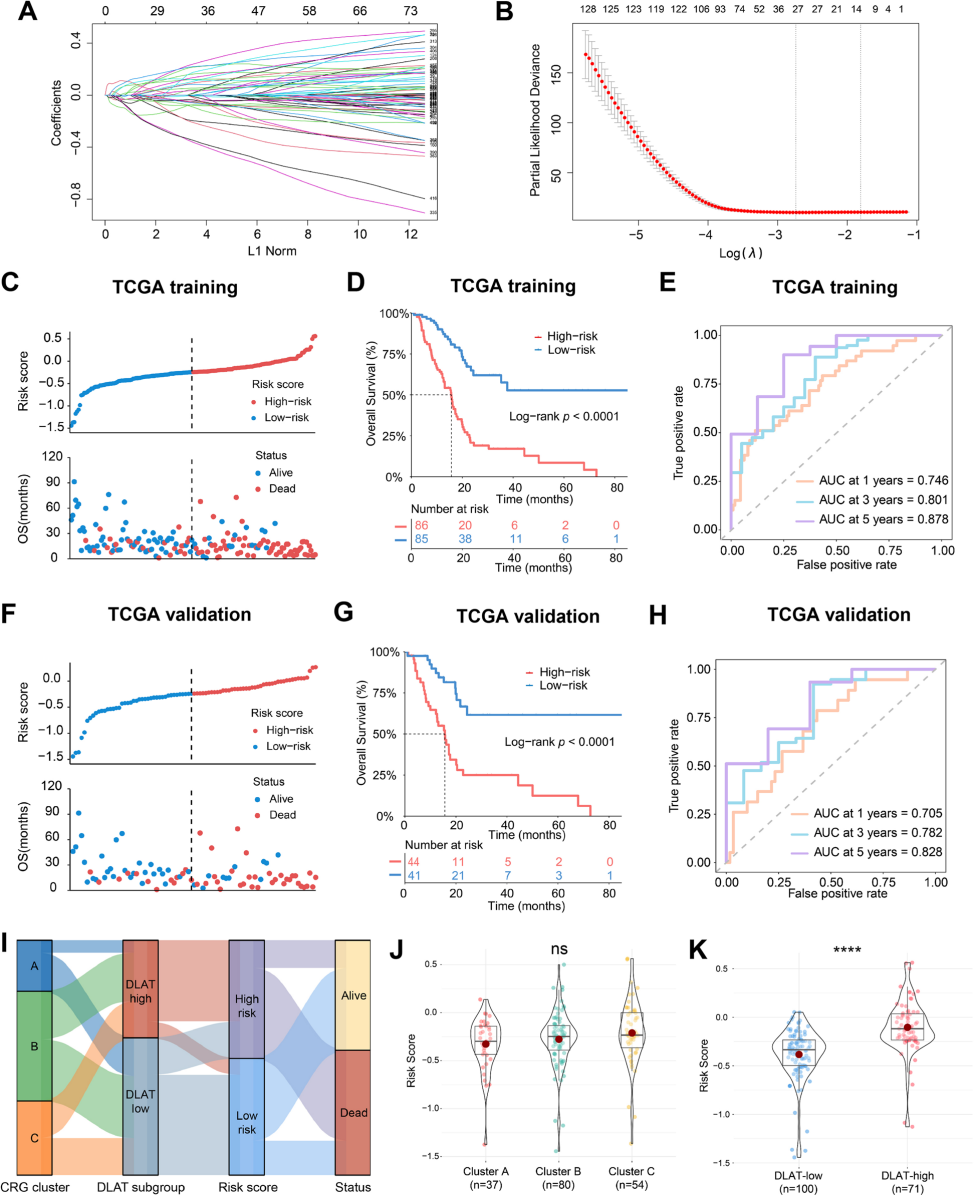
Construction and validation of risk score model. **A**,** B **Thirteen modeling genes determined by LASSO Cox regression. **C** Distribution of risk score and survival status in TCGA training cohort. **D** Kaplan–Meier curves for high-risk and low-risk patients in TCGA training cohort. **E** Time-dependent ROC curves for predicting the 1-, 3-, and 5-year survival status in TCGA training cohort. **F** Distribution of risk score, **G** Kaplan–Meier curves, and **H** Time-dependent ROC curves in TCGA validation cohort. **I** Sankey diagram of different CRG clusters, DLAT subgroups, risk scores, and survival outcomes. **J** Comparison of risk scores in cluster A, B, and C. **K** Comparison of risk scores between DLAT-high and DLAT-low groups (ns represents no significance, ****p < 0.0001)

To create the DLAT-based model, we defined 427 prognostic DEGs by univariate Cox regression analysis (Supplementary Table 4) and selected 13 optimal genes through LASSO regression analysis (Fig. 8A-B). According to the 13-gene model, patients were scored using the following formula and then split into high-risk and low-risk groups based on the median risk score. Risk score = TSPOAP1 * (-0.02367876) + ANLN * 0.011937625 + TLE2 * (-0.08933642) + GNB3 * (-0.036416417) + GLTPD2 * (-0.018670796) + BPIFB4 * (-0.003192766) + FAM111B * 0.102912035 + INSYN2B * 0.070370212 + RNU6-892P * (-0.017035372) + LINC01940 * 0.044119945 + ANKRD18B * 0.034310234 + CCDC188 * (-0.012306307) + HNRNPD-DT * (-0.037777293).

The risk score increased along with the death rates in the training cohort (the whole dataset, *n =* 171) (Fig. 8C). Moreover, the OS was significantly prolonged in low-risk group (log-rank test, *p* < 0.0001) (Fig. 8D). The ROC analysis demonstrated that this model had a high accuracy in predicting 1-, 3-, 5- year survival status, with the AUC values of 0.746, 0.801, and 0.878, respectively (Fig. 8E). Additionally, this risk model performed well in the validation cohort (randomly selected, *n =* 85) (Fig. 8F-H).

The Sankey diagram displayed the proportions of patients with different CRG clusters, DLAT expression, risk scores, and survival outcomes (Fig. 8I). Patients in cluster C showed higher risk scores than those in other clusters, however, the difference was not significant (Fig. 8J). Furthermore, DLAT-high patients exhibited statistically higher risk scores than DLAT-low patients (Fig. 8K).

### Validation of the expression of DLAT in PAAD

**Fig. 9:**
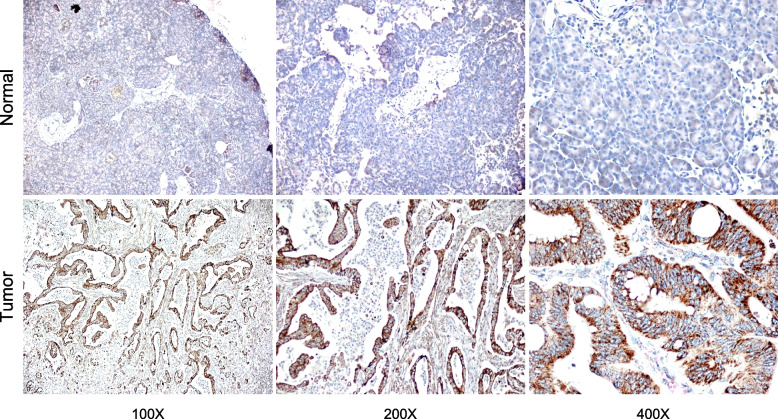
Representative IHC images of DLAT staining in pancreatic tumor and adjacent normal tissues

We performed RT-qPCR to further confirm the differential expression of DLAT. The results exhibited that DLAT mRNA was significantly increased in tumor cell lines compared to normal cell line (Supplementary Fig. 2). Moreover, we collected pancreatic tumor tissues and adjacent normal tissues from 12 patients. Their clinical characteristics were summarized in Supplementary Table 5. The IHC assays demonstrated that DLAT was highly expressed in pancreatic cancer tissues (Fig. 9).

## Discussion

PAAD is a common malignant tumor worldwide with an augmented incidence over the past decades. Despite advances in the treatment of PAAD, there was very little improvement in OS, with a 5-year survival rate of 9% [41]. Thus, it is ultimate to discover novel therapeutic targets to improve the clinical outcomes of PAAD patients.

Tsvetkov et al. revealed a novel cell death pathway termed "cuproptosis" induced by intracellular copper. Unlike other forms, cuproptosis occurs when excessive copper ions bind to mitochondrial lipoylated components, causing cell death by proteotoxic stress. However, the role that cuproptosis plays in PAAD remains to be illuminated.

In this work, we carried out the bioinformatic analysis of CRGs using public data acquired from TCGA, GTEx, and GEO databases. We examined the expression of CRGs and found that most genes exhibited increased expression levels in PAAD tissues. Subsequently, patients were classified into 3 subgroups using consensus clustering analysis. Patients in cluster A showed the most favorable behaviors with the longest OS time. The enrichment analysis indicated that upregulated DEGs in cluster A were mainly enriched in cellular metabolism pathways (such as retinol metabolism, drug metabolism by cytochrome P450, and xenobiotics metabolism by cytochrome P450), and tumor-related signaling pathways (such as Wnt/β catenin pathways, DNA repair, p53 pathways, and unfolded protein response). Previous researches have fully uncovered that these pathways played a significant role in cancer development by regulating cellular metabolism [42], cell growth [43], apoptosis [44], and tumor immunity [45]. Accordingly, CRGs may participate in the development of PAAD via these classical pathways.

The "Warburg effect" is a unique metabolic feature in tumors, that is, the energy required for cancer cellular processes is primarily generated by aerobic glycolysis rather than mitochondrial oxidative phosphorylation [46]. A newly developed viewpoint showed that the changes in tumor metabolism may be partly connected to impaired mitochondrial function caused by the inhibition of pyruvate dehydrogenase complex (PDC) [47]. PDC is an enzyme complex responsible to regulate glucose oxidative metabolism. It is composed of pyruvate dehydrogenase (E1), dihydrolipoamide acetyltransferase (E2), and dihydrolipoamide dehydrogenase (E3) [48]. These components work orderly in the inner mitochondrial membrane to catalyze the conversion of oxidative decarboxylation of pyruvate to acetyl coenzyme A, thereby interconnecting glycolysis and TCA cycle. Nevertheless, the inactivation of PDC controlled by pyruvate dehydrogenase kinase limits the synthesis of acetyl coenzyme A, so that glucose oxidation is blocked and metabolic disorders are developed [49].

The dihydrolipoamide acetyltransferase (PDC-E2) is encoded by DLAT gene. Dihydrolipoamide acetyltransferase has been widely described in an autoimmune liver disease, primary biliary cholangitis, in which patients lost their immune tolerance to PDC-E2 [50, 51]. Autoreactive T lymphocytes were increased in primary biliary cholangitis and destroyed bile duct epithelial cells, leading to destructive lymphocytic cholangitis, ultimately resulting in cirrhosis and even liver failure. Moreover, pyruvate dehydrogenase deficiency, a disease characterized by primary lactic acidosis and neurological dysfunction, was related to DLAT gene mutation [52]. As for its role in tumor development, Shan et al. reported that DLAT emerged as an upstream acetyltransferase for K76 and activated oxidative pentose phosphate pathway, finally promoting cell growth in cancer cells [53]. In addition, DLAT was increased in gastric cancer, and siRNA-mediated knockdown of DLAT could increase the pyruvate levels [54]. DLAT-mediated glycolysis reprograming also contributed to the carcinogenesis of non-small cell lung cancer [55]. Furthermore, DLAT exhibited prognostic values in hepatocellular carcinoma patients based on public databases and clinical samples [56].

Although CRGs have been studied in PAAD, DLAT has hardly been considered as a special gene for systemic analysis. Researchers generally analyzed the CRGs as a whole rather than focusing on one gene [57–60]. Compared to other genes, DLAT possessed the lowest *p* value in prognostic analysis, indicating that it may play a more important role in the prognosis of PAAD. Therefore, a comprehensive analysis of DLAT was needed. In this work, we found that DLAT served as an independent risk factor for OS in PAAD. KEGG pathway analysis revealed that DLAT was primarily correlated to TCA cycle and pancreatic cancer pathways, which was in accordance with the fact that cuproptosis was related to lipoylated TCA cycle proteins, further demonstrating the rationality and feasibility of our work. Functional enrichment analysis also revealed that DLAT was engaged in cell division, indicating that DLAT might promote tumor progression by activating proliferative processes, such as mismatch repair, DNA replication, nucleotide excision repair, and cell cycle.

As an essential trace element, copper is required for maintaining enzyme functions and body homeostasis [13]. The levels of copper were usually increased in malignant tumors [61, 62]. Copper was involved in the regulation of cellular and humoral immunity, and both excessive or insufficient copper ions will impair cellular functions [63, 64]. We evaluated the relationship between DLAT and multiple immunological characteristics in PAAD. As a result, DLAT showed significantly positive correlations with immune cell infiltration, most cancer-immunity cycle steps, multiple immunotherapy-predicted pathways, and common inhibitory immune checkpoints. Voli et al. reported that copper could enhance PD-L1 expression and promote cancer immune evasion in vitro, while copper chelators could increase CD8 + T cell infiltration and prolong survival time in vivo [65]. Similarly, DLAT-high patients with increased PD-L1 expression had worse survival status than DLAT-low patients. Furthermore, DLAT-high patients were more sensitive to respond to anti-CTLA-4/PD-L1 treatment than DLAT-low patients, implying that DLAT may function as a biomarker to predict immunotherapy response.

In actual clinical practice, with the help of risk score model, doctors can better comprehend the status of patients to make precise treatment decisions. Since the introduction of cuproptosis, many researchers have developed the risk score models to predict the clinical outcomes of patients with PAAD. However, the reported models exhibited relatively lower AUC values (range from 0.68–0.84) [57–59]. Therefore, a more effective model was needed. Considering the potential effect of DLAT on clinical outcomes, we constructed a risk model based on DLAT to assess the individual survival risks. It was found that the model we built showed higher AUC values than others (Supplementary Table 6). Thus, the DLAT-based model possessed better effectiveness in predicting prognosis and could be more useful in clinical practice.

Overall, we comprehensively evaluated the prognostic values and biological function of DLAT in PAAD. In addition, we revealed the relationship between DLAT and multiple immunological characteristics, which could provide new ideas for tumor immunotherapy. We also constructed a DLAT-based model to predict patients’ prognosis. However, our study still has certain limitations. Firstly, the prognostic and immunotherapeutic implications of DLAT were analyzed using public databases, which may cause a certain bias. Secondly, the impact of DLAT on tumor progression needs to be further validated by cell function experiments. In conclusion, more experimental and clinical data are required in the future to verify the above conclusions.

## Conclusion

We conducted a systematic analysis of DLAT in PAAD by integrated bioinformatic methods and clinical validation. It was identified that DLAT could emerge as an independent risk factor for survival, with the potential to guide personalized immunotherapy in PAAD. Moreover, the DLAT-based model exhibited high accuracy in prognosis prediction. Our findings could help illuminate the function of DLAT and contribute to accurate treatment of PAAD.

## Supplementary Information

**Additional file 1:** **Supplementary Table 1. **Correlation between DLAT and other genes in pancreatic cancer. **Supplementary Table 2. **GSEA of DLAT co-expression network based on "HALLMARK" gene sets. **Supplementary Table 3.** DEGs between DLAT-high and DLAT-low groups. **Supplementary Table 4.** Univariate Cox regression analysis identified 427 prognostic DEGs between DLAT-high and DLAT-low groups. **Supplementary Table 5.** Clinical characteristics of 12 pancreatic cancer patients. **Supplementary Table 6.** Comparison of AUC values between DLAT-based model and other models.

**Additinal file 2:** **Supplementary Figure 1. **Prediction of immunotherapy response differences between DLAT-high and DLAT-low groups in (A) IMvigor210, (B) CheckMate, (C) GSE78220, and (D) GSE91061 cohorts (R represents responsive, while noR represents no responsive). (E) Correlation analysis between DLAT and PD-L1, CTLA-4, as well as PD-1. **Supplementary Figure 2. **The mRNA expression of DLAT in pancreatic cancer cell lines (BxPC-3 and PANC-1) and normal cell line (HPDE6-C7) (***p < 0.001).

## Data Availability

RNA-sequencing data can be obtained from the UCSC Xena (https://xenabrowser.net/datapages/) (GDC TCGA PAAD and GTEx). The study’s GEO database (GSE62452, GSE71729, GSE15471, GSE16515, GSE78220, and GSE91061) can be downloaded from https://www.ncbi.nlm.nih.gov/gds/. The original data presented in the study were included in Supplementary Information. Further inquiries can be directed to the corresponding author.

## Abbreviations

PAAD: Pancreatic adenocarcinoma
CRGs: Cuproptosis-related genes
DLAT: Dihydrolipoamide acetyltransferase
RT-qPCR: Quantitative reverse transcriptase polymerase chain reaction
IHC: Immunohistochemistry
PD-1: Programmed cell death-1
PD-L1: Programmed cell death-ligand 1
CTLA-4: Cytotoxic T-lymphocyte antigen-4
TME: Tumor microenvironment
TCA: Tricarboxylic acid
PPI: Protein-protein interaction
DEGs: Differentially expressed genes
TCGA: The Cancer Genome Atlas
GTEx: Genotype-tissue expression
GEO: Gene Expression Omnibus
FPKM: Fragments per kilobase million
TPM: Transcripts per million
CNV: Copy number variation
GO: Gene ontology
KEGG: Kyoto Encyclopedia of Genes and Genomes
GSEA: Gene set enrichment analysis
GSVA: Gene set variation analysis
ssGSEA: Single sample gene set enrichment analysis
OS: Overall survival
HR: Hazard ratio
CI: Confidence interval
ICB: Immune checkpoint blockade
ROC: Receiver operating characteristic
AUC: Areas under the curve
PDC: Pyruvate dehydrogenase complex
