# Supplementary material for: Comprehensive analysis identifies cuproptosis-related gene DLAT as a potential prognostic and immunological biomarker in pancreatic adenocarcinoma
Source: BMC Cancer. 2023 Jun 17;23:560. doi: 10.1186/s12885-023-11042-7 (PMC10276918; doi:10.1186/s12885-023-11042-7)
Supplement: Supplementary file 2 — Additinal file 2: Supplementary Figure 1. Prediction of immunotherapy response differences between DLAT-high and DLAT-low groups in (A) IMvigor210, (B) CheckMate, (C) GSE78220, and (D) GSE91061 cohorts (R represents responsive, while noR represents no responsive). (E) Correlation analysis between DLAT and PD-L1, CTLA-4, as well as PD-1. Supplementary Figure 2. The mRNA expression of DLAT in pancreatic cancer cell lines (BxPC-3 and PANC-1) and normal cell line (HPDE6-C7) (***p < 0.001). [file 12885_2023_11042_MOESM2_ESM.docx]

**Supplementary Information**

**Comprehensive analysis identifies cuproptosis-related gene DLAT as a potential prognostic and immunological biomarker in pancreatic adenocarcinoma**

Xiaoling Zhang^1†^, Yuxin Zhou^1†^, Jiahe Hu^1†^, Xuefeng Yu^2^, Haitao Xu^3^, Zhichang Ba^4^, Haoxin Zhang^1^, Yanan Sun^1^, Rongfang Wang^1^, Xinlian Du^1^, Ruishu Mou^1^, Xuedong Li^1^, Jiuxin Zhu^5*^, Rui Xie^1*^

^1^ Department of Digestive Internal Medicine, Harbin Medical University Cancer Hospital, Harbin 150081, China

^2^ Department of Gastroenterological Surgery, Harbin Medical University Cancer Hospital, Harbin 150081, China

^3^ Department of Hepatobiliary and Pancreatic Surgery, Harbin Medical University Cancer Hospital, Harbin 150081, China

^4^ Medical Imaging Center, Harbin Medical University Cancer Hospital, Harbin 150081, China

^5^ Department of Pharmacology (State-Province Key Laboratories of Biomedicine-Pharmaceutics of China, Key Laboratory of Cardiovascular Medicine Research, Ministry of Education), College of Pharmacy, Harbin Medical University, Harbin 150081, China

^†^Xiaoling Zhang, Yuxin Zhou, and Jiahe Hu contributed equally to this work.


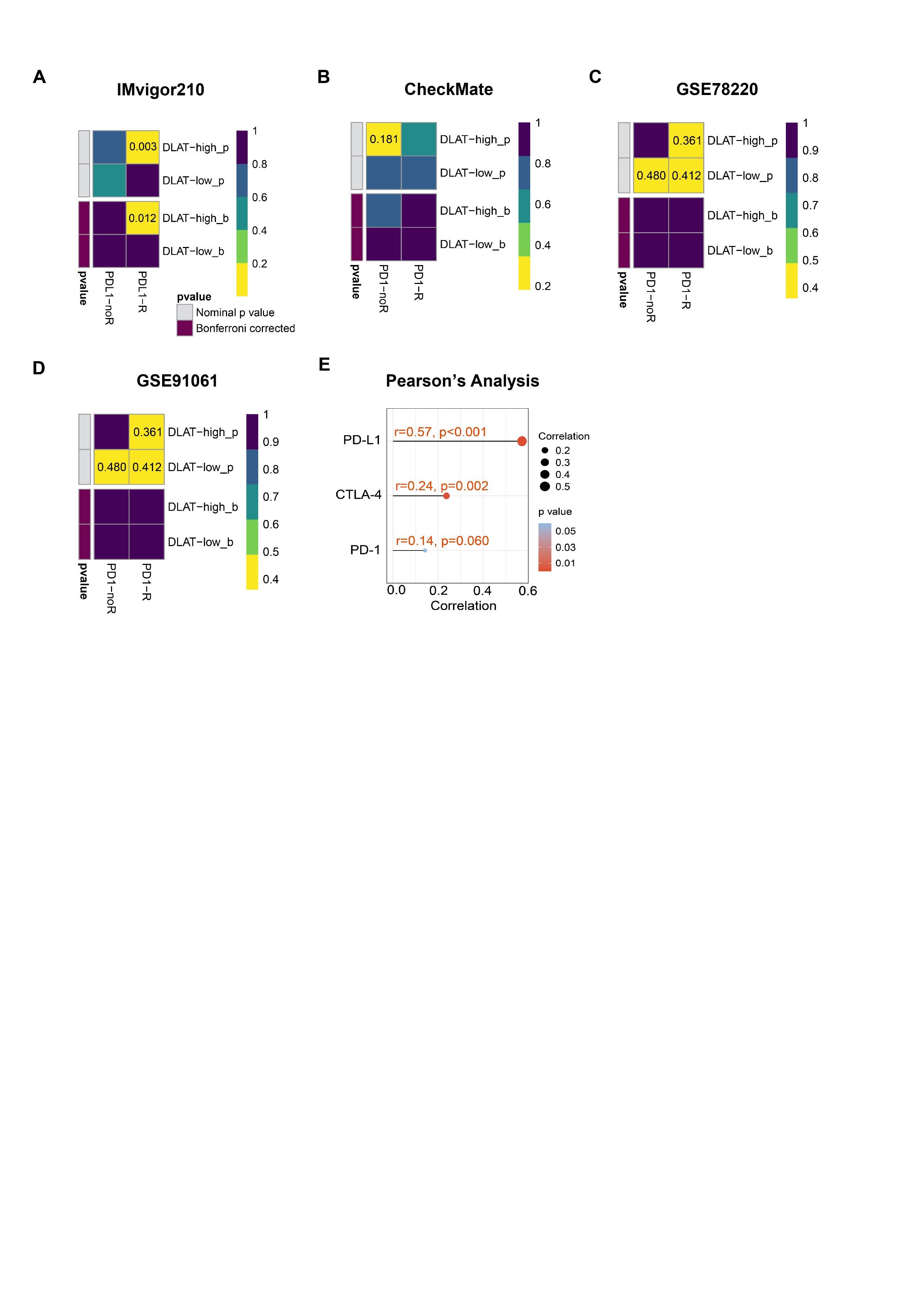


**Supplementary Figure 1.** Prediction of immunotherapy response differences between DLAT-high and DLAT-low groups in **(A)** IMvigor210, **(B)** CheckMate, **(C)** GSE78220, and **(D)** GSE91061 cohorts (R represents responsive, while noR represents no responsive). **(E)** Correlation analysis between DLAT and PD-L1, CTLA-4, as well as PD-1.


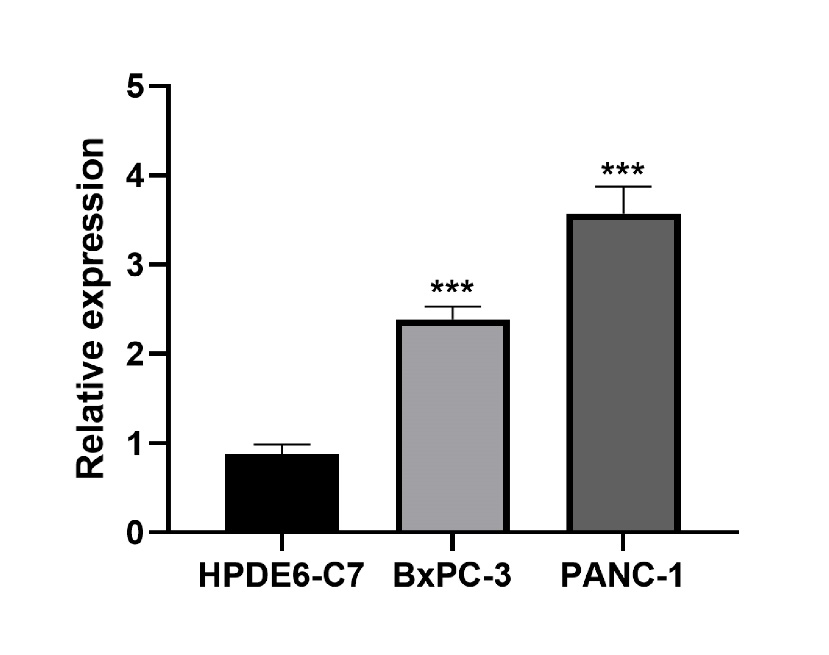


**Supplementary Figure 2.** The mRNA expression of DLAT in pancreatic cancer cell lines (BxPC-3 and PANC-1) and normal cell line (HPDE6-C7) (****p* < 0.001).
